# Prevalence of gastrointestinal parasites in domestic cats (*Felis catus*) diagnosed by different coproparasitological techniques in the municipality of Seropédica, Rio de Janeiro

**DOI:** 10.1590/S1984-29612023049

**Published:** 2023-08-11

**Authors:** Ygor Henrique da Silva, Diefrey Ribeiro Campos, Gabriel Alcides Capucho Lima, Janaína Pires Quintal, Brena Gava Guimarães, Guilherme Mota Maciel do Rêgo, Barbara Rauta de Avelar, Juliana de Moraes Intrieri, Thais Ribeiro Correia, Fabio Barbour Scott

**Affiliations:** 1 Departamento de Parasitologia Animal, Instituto de Veterinária, Universidade Federal Rural do Rio de Janeiro - UFRRJ, Seropédica, RJ, Brasil

**Keywords:** Feline, fecal examination, helminth, protozoan, epidemiology, Felino, examinação fecal, helminto, protozoário, epidemiologia

## Abstract

The objectives of this work were to investigate the occurrence of parasites in feces of cats, compare different coproparasitological techniques for their diagnosis and determine associations with parasitism. The samples were processed using three different coproparasitological techniques: centrifugal flotation in sucrose, centrifugal flotation in ZnSO_4_ and simple sedimentation. The parasitic association between parasitism and variables such as age, sex and fecal consistency was performed using the chi-square test or the G test with a significance level of 5%. A total of 237 samples were analyzed, of which 93 (39.2%) were positive, being *Ancylostoma* spp. (17.3%), *Giardia intestinalis* (12.2%), *Platynosomum illiciens* (8.0%), *Cystoisospora* spp. (6.3%), *Toxoplasma gondii/ Hammondia hammondi* (3.4%), Diphyllobothriidae (2.1%), *Toxocara* spp. (1.7%), *Dipylidium caninum* (1.3%) and *Mesocestoides* spp. (0.8%). In the parasitism association analysis, it was possible to verify a statistical difference in the age category for *Cystoisospora* spp. (*p*=0.001) observing a strong relationship between parasitism and young animals, the association with sex proved to be important for *P. illiciens* (*p*<0.001) with a higher frequency of parasitized females and fecal consistency revealed to be related to the parasites *G. intestinalis* (*p*=0.007) and *P. illici*ens (*p*=0.033) showing a higher number of positive animals for these parasites with normal fecal consistency. In conclusion, we observed a higher occurrence of *Ancylostoma* spp. and *G. intestinalis* in fecal samples from domestic cats received in routine diagnoses and the presence of other parasites with zoonotic potential, as well as the relationship of these diagnosed parasites with the categories sex, age and fecal consistency.

## Resumo

Os objetivos deste trabalho foram investigar a ocorrência de parasitos em fezes de gatos, comparar diferentes técnicas coproparasitológicas para o seu diagnóstico e determinar as associações com o parasitismo. As amostras foram processadas usando três diferentes técnicas coproparasitológicas: centrífugo-flutuação em sacarose, centrífugo-flutuação em ZnSO_4_ e sedimentação simples. A associação parasitária entre o parasitismo e variáveis, como idade, sexo e consistência fecal, foi realizada pelo teste de qui-quadrado ou teste de G com nível de significância de 5%. No total, foram analisadas 237 amostras, das quais 93 (39,2%) foram positivas, sendo diagnosticados os parasitos *Ancylostoma* spp. (17,3%), *Giardia intestinalis* (12,2%), *Platynosomum illiciens* (8,0%), *Cystoisospora* spp. (6,3%), *Toxoplasma gondii*/ *Hammondia hammondi* (3,4%), Diphyllobothriidae (2,1%), *Toxocara* spp. (1,7%), *Dipylidium caninum* (1,3%) e *Mesocestoides* spp. (0,8%). Na análise de associação entre o parasitismo e as outras variáveis, foi possível verificar diferença estatística na categoria idade para *Cystoisospora* spp. (*p*=0,001), observando forte relação entre o parasitismo e animais jovens. A associação com o sexo demonstrou ser importante para *P. illiciens* (*p*<0,001), com maior frequência de fêmeas parasitadas. E a consistência fecal revelou ter relação com os parasitos *G. intestinalis* (*p*=0,007) e *P. illiciens* (*p*=0,033), com maior frequência de fezes com consistência normal nos animais positivos para esses parasitos. Em conclusão, foi observada uma maior ocorrência de *Ancylostoma* spp. e *G. intestinalis* em amostras fecais de gatos domésticos, recebidas em diagnósticos de rotina e a presença de outros parasitos com potencial zoonótico, como também, a relação desses parasitos diagnosticados com as categorias sexo, idade e consistência fecal.

## Introduction

In the 19th century, during the Victorian era, cats were brought into homes and treated as domestic animals (Serpell, 2000). With the increase in urbanization and the verticalization of cities, cats are now considered good companion animals because they adapt better to small spaces. During the coronavirus disease 2019 (COVID-19) pandemic, the number of pet cats in Brazil showed a cumulative increase of 5.9% between 2020 and 2021 (ABINPET, 2022).

Pets can provide their owners with emotional comfort (Giumelli & Santos, 2016); however, it is important to emphasize that the increase in human-animal contact, when carried out without adequate instruction, can lead to the maintenance and transmission of zoonotic pathogens, such as parasites (Crowley et al., 2019).

Cats can be affected by several parasitic diseases that impact human and animal health. Some parasites affect the digestive system, such as nematodes *Ancylostoma* spp. and *Toxocara* spp., the etiological agents of cutaneous larva migrans and visceral larva migrans. Protozoa such as *Giardia intestinalis* and *Cryptosporidium* spp. are responsible for enteric disorders (Ferreira et al., 2013), in addition to *Toxoplasma gondii*, which can cause important alterations during the fetal period in humans and other mammals (Souza et al., 2017; Ubirajara et al., 2022).

In addition to their importance in public health, parasites of the cat digestive system are related to important diseases such as gastroenteritis caused by *Ancylostoma* spp., *Toxocara* spp., *G. intestinalis*, *Cystoisospora* spp., and *Cryptosporidium* spp., and parasitic cholangitis caused by the trematode *Platynosomum illiciens*. All these diseases, when not diagnosed and treated correctly, have the potential to lead to animal death (Bowman et al., 2002; López et al., 2006).

The key to reducing the transmission of these diseases from cats to humans is proper diagnosis for establishing a correct treatment. In this context, epidemiological studies and evaluations of diagnostic techniques help to better understand the prevalence of these parasites and guide clinicians (Campos et al., 2016; Mandarino-Pereira et al., 2010). This study aimed to report the frequency of parasites identified in coproparasitological examinations of domestic cats, belonging to the routine parasitology, between 2020 and 2021, associated with parasitism, age, sex, and fecal consistency, using three different parasitological techniques.

## Materials and Methods

### Study location

This study was conducted in the municipality of Seropédica, RJ.

### Samples

Fecal samples from domestic cats (*Felis catus*) obtained from the coproparasitological routine requested by veterinarians between September 2020 and November 2021 were analyzed using three laboratory techniques commonly used for the diagnosis of parasitic structures (eggs, cysts, and oocysts). The samples were stored in a refrigerator (2-8 °C) until processing, not exceeding 24 hours after receipt so as not to compromise the diagnosis.

The fecal consistency of each sample was assessed using an adaptation of the fecal scoring system for cats described by German et al. (2017), with scores from one to six; fecal consistency increased according to the score, ranging from diarrheal to dry stools, with scores of three and four being normal. Data on the animals, such as name, species, breed, age, and sex, were obtained from the Parasitological Examination Requisition forms. Age-related data were grouped and defined as follows: young animals <1 year old, adult animals 1-8 years old, and senior animals >8 years old.

### Coproparasitological techniques

All fecal samples were subjected to three coproparasitological techniques and evaluated for the presence of parasitic forms. The techniques used were centrifugal flotation in sucrose (CFS); density: 1.28 g/mL (Sheather, 1923), centrifugal flotation on zinc sulfate (ZnSO_4_); density: 1.18 g/mL (Faust et al., 1939), and spontaneous sedimentation (Hoffman et al., 1934). The diagnosis of parasitic structures was based on the morphological characteristics reported by Soulsby (1987) and Zajac & Conboy (2012), and the samples were photodocumented by light microscopy with the aid of the Future Win Joe software program (Software.informer, 2022).

CFS is not the technique of choice for diagnosing *G. intestinalis* cysts or *T. gondii/H. hammondi* oocysts, and because some artifacts generated made diagnosis difficult owing to their high density, they were not used for the diagnosis of these parasites.

### Data analysis

The collected data were analyzed using descriptive statistics, with simple frequencies for qualitative variables and measures of central tendencies for quantitative variables. The association between techniques, fecal consistency, age, sex and parasitism was evaluated using the chi-square test for two variables and the G test for three or more variables. All statistical analyses were performed using the BioEstat 5.3 computer program with a 95% confidence interval (*p* < 0.05) (Ayres et al., 2007).

## Results

In total, 237 fecal samples were evaluated, and the identification of any parasitic structure in at least one of the evaluated coproparasitological techniques was considered positive, with a percentage occurrence of 39.2% (93/237) of positive samples (Table 1).

**Table 1:** Frequency of parasites diagnosed in coproparasitological examinations of cats during routine parasitology diagnoses between the years 2020 and 2021.

**Parasites**		**No. positive/ No. Sampled (% positive)**
**Nematodes**		
	*Ancylostoma* spp.	41/237 (17.3)
	*Toxocara* spp.	4/237 (1.7)
**Cestodes**		
	Diphyllobothriidae	5/237 (2.1)
	*Dipylidium caninum*	3/237 (1.3)
	*Mesocestoides* spp.	2/237 (0.8)
**Trematode**		
	*Platynosomum illiciens*	19/237 (8.0)
**Protozoa**		
	*Giardia inteslinalis*	29/237 (12.2)
	*Cystoisospora* spp.	15/ 237 (6.3)
	*Toxoplasma gondii/ Hammondia hammondi*	8/237 (3.4)
	**Total**	**93/237 (39.2)**

**Figure 1:**
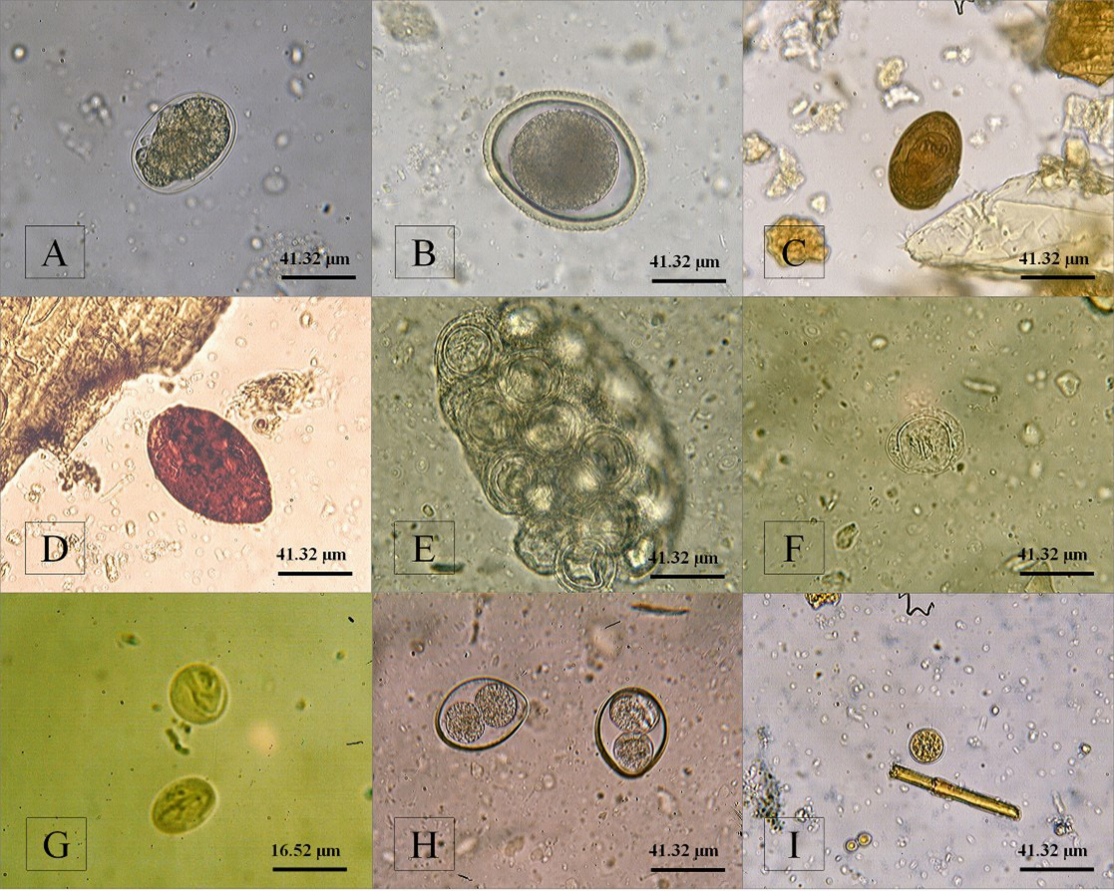
Parasitic forms (eggs, cysts and oocysts) observed in coproparasitological examinations of cats. (A) Egg of *Ancylostoma* spp. (B) Egg of *Toxocara* spp. (C) Egg of *Platynosomum illiciens* (D) Egg of the family Diphyllobothriidae stained with lugol. (E) Ovigerous capsule of *Dipylidium caninum*. (F) Egg of *Mesocestoides* spp. (G) Cysts of *Giardia intestinalis* stained with Lugol. (H) Oocysts of *Cystoisospora* spp. (I) Unsporulated oocyst of *Toxoplasma gondii/Hammondia hammondi* stained with Lugol.

Among the diagnosed parasites, a higher occurrence of animals that tested positive for *Ancylostoma* spp. (17.3%) was observed, followed by *G. intestinalis* (12.2%), *P. illiciens* (8.0%), and *Cystoisospora* spp. (6.3%) (Table 1). However, the structures of other parasites, such as *Toxocara* spp., *D. caninum*, *Mesocestoides* spp., *T. gondii/ H. hammondi*, and diphyllobothrids, were also observed but less frequently (Table 1; Figure 1).

Of the 93 positive samples, 66 (70.97%) showed parasitism for a single species, genus, or family of parasites, and 27 (29.03%) samples showed multiparasitism (two or more). Still in this context, the parasites *Ancylostoma* spp., *G. intestinalis*, and *P. illiciens* were the most frequently found in association, as shown in Table 2.

**Table 2:** List of parasites and their associations in coproparasitological diagnosis of fecal samples from domestic cats.

**Parasites**	**No. positive/ No. Sampled (% positive)**
*Ancylostoma* spp.	24/93 (25.81)
*Cystoisospora* spp.	10/93 (10.75)
Diphyllobothriidae	1/93 (1.08)
*Dipylidium caninum*	1/93 (1.08)
*Giardia intestinalis*	15/39 (16.13)
*Mesocestoides* spp.	2/93 (2.15)
*Platynosomum illiciens*	9/93 (9.68)
*Toxocara* spp.	3/93 (3.23)
*Toxoplasma gondii/ Hammondia hammondi*	1/93 (1.08)
*Ancylostoma* spp. + *Cystoisospora* spp.	2/93 (2.15)
*Ancylostoma* spp. + *Cystoisospora* spp. + *Giardia intestinalis*	1/93 (1.08)
*Ancylostoma* spp. + *Giardia intestinalis*	6/93 (6.45)
*Ancylostoma* spp. + *Giardia intestinalis* + Diphyllobothriidae	1/93 (1.08)
*Ancylostoma* spp. + *Platynosomum illiciens*	3/93 (3.23)
*Ancylostoma* spp. + *Platynosomum illiciens* + Diphyllobothriidae + *Toxoplasma gondii/ Hammondia hammondi*	1/93 (1.08)
*Ancylostoma* spp. + *Platynosomum illiciens* + *Giardia intestinalis*	1/93 (1.08)
*Ancylostoma* spp. + *Toxoplasma gondii/ Hammondia hammondi*	2/93 (2.15)
*Cystoisospora* spp. + Diphyllobothriidae	1/93 (1.08)
*Cystoisospora* spp. + *Giardia intestinalis*	1/93 (1.08)
Diphyllobothriidae + *Platynosomum illiciens*	1/93 (1.08)
*Dipylidium caninum* + *Toxoplasma gondii/ Hammondia hammondi*	1/93 (1.08)
*Giardia intestinalis* + *Toxoplasma gondii/ Hammondia hammondi*	1/93 (1.08)
*Platynosomum illiciens* + *Giardia intestinalis*	2/93 (2.15)
*Platynosomum illiciens* + *Dipylidium caninum*	1/93 (1.08)
*Platynosomum illiciens* + *Toxoplasma gondii/ Hammondia hammondi*	1/93 (1.08)
*Toxocara* spp. + *Giardia intestinalis* + *Toxoplasma gondii/ Hammondia hammondi*	1/93 (1.08)
**Total**	**93/93 (100)**

Regarding the positive animals independent of the parasite in the evaluated categories, in the sex category, 35.5% (33/93) males and 64.5% (60/93) positive females were observed. In the age category, 30.7% (28/93) were young cats, 63.74% (58/93) were adults and 5.49% (5/93) were senior cats. And in the category of fecal consistency, 45.16% (42/93) and 27.96% (26/93) of the parasitized animals had fecal consistency of type 3 and 4, respectively. However, the statistical analysis revealed a significant difference only in the age category (*p* = 0.0267).

Performing the analysis separately for sex, age, and fecal consistency categories by parasite, no differences were observed between animals parasitized by the nematodes *Ancylostoma* spp. and *Toxocara* spp. For the trematode *P. illiciens*, differences were observed in sex (*p* < 0.0001) and fecal consistency (*p* = 0.033) categories.

Regarding the diagnosed cestodes, no statistical differences were observed when comparing positive animals according to sex, age, or fecal consistency.

As for the protozoa diagnosed in the present study compared to the categories of sex, age, and fecal consistency, statistical differences were observed in relation to the fecal consistency of animals parasitized by *G. intestinalis* (*p* = 0.007) and for individuals in the age category positive for *Cystoisospora* spp. (*p* = 0.001).

Comparison of the number of diagnoses between the techniques did not show significant differences (*p* = 0.4685). However, when related to the parasites, significance was observed for *P. illiciens* (*p* = 0.002), *Cystoisospora* spp. (*p* = 0.0461) and Diphyllobothriidae (*p* = 0.0076) (Table 3).

**Table 3:** Frequency of gastrointestinal parasites detected in fecal samples of domestic cats using three coproparasitological techniques.

	**Parasites**	**CFS**	**Faust**	**HPJ**	***p-value*** +
Nematodes	*Ancylostoma* spp.	39 (16.5%)	32 (13.5%)	22 (9.3%)	0.0634
	*Toxocara* spp.	4 (1.7%)	4 (1.7%)	4 (1.7%)	1
Cestodes	Diphyllobothriidae	0 (0.0%)	0 (0.0%)	5 (2.1%)	0.0076*
	*Dipylidium caninum*	3 (1.3%)	0 (0.0%)	1 (0.4%)	0.1577
	*Mesocestoides* spp.	2 (0.8%)	0 (0.0%)	0 (0.0%)	0.1916
Trematode	*Platynosonum illiciens*	15 (6.3%)	2 (0.8%)	12 (5.1%)	0.002*
Protozoa	*Giardia intestinalis*	-	28 (11.8%)	15 (6.3%)	0.0535
	*Cystoisospora* spp.	10 (4.2%)	8 (3.4%)	2 (0.8%)	0.0461*
	*Toxoplasma gondii/ Hammondia hammondi*	-	1 (0.4%)	7 (3.0%)	0.0643
	**Total**	**66 (27.8%)**	**64 (27%)**	**55 (23.2%)**	**0.4685**

CFS = Centrifugal flotation technique in sucrose; Faust = Centrifugal-flotation technique in ZnSO_4_; HPJ = technique of Hoffman, Pons and Janer, 1934.

^+^ = p values from G test;

* *p*-value < 0.05 indicating significant difference.

## Discussion

Studies related to the prevalence of gastrointestinal tract parasites in domestic animals are important because they demonstrate the frequency of different groups of parasites in a given location, allowing for the evaluation of the main clinical signs and the development of strategies to control or prevent these parasites (Labarthe et al., 2004). Analysis of 237 fecal samples from domestic cats using the three coproparasitological techniques revealed that 93 (39.2%) samples were positive for one or more parasitic structures, observing a higher frequency of parasites such as *Ancylostoma* spp., *G. intestinalis*, *P. illiciens* and *Cystoisospora* spp. This occurrence can be classified as moderate, as the prevalence in recent coproparasitological studies in cats in the country varied between 17% and 75.26% (Campos et al., 2016; Lima et al., 2018; Quadros et al., 2021).

However, both the prevalence of parasitized animals and the frequency of parasites reported in epidemiological studies are influenced by factors such as the population number, age and sex of the animals, whether or not they are domiciled (Lima et al., 2018), the load of parasitic infection and the type of diagnostic technique used (Monteiro et al., 2016), requiring robust studies to better understand this inconstancy.

Despite the different factors that influence the frequency of certain parasites, many authors have reported the nematode *Ancylostoma* spp. as the main parasite diagnosed and the one most associated with mixed infections with other parasites of the gastrointestinal tract (Rezende et al., 2015; Campos et al., 2016; Monteiro et al., 2016). Of the 93 animals that tested positive in the present study, 27 (29.03%) were positive for two or more species/genera or families of parasites. In addition to *Ancylostoma* spp., *G. intestinalis* and *P. illiciens* were most frequently associated with other parasites in coproparasitological examinations, demonstrating that other parasites were also frequent in coinfections.

The correlation of the presence or absence of parasitism in relation to the categories sex, age, and type of fecal consistency showed significance only in relation to the age of infected individuals (*p* = 0.0267), suggesting that young individuals are more likely to be infected when compared to the other categories. This result may be explained by the fact that young animals do not have previous exposure to parasitism and have an immature immune system, which allows for greater susceptibility and acceptability to infection (Carrasco et al., 2016).

Comparing the categories evaluated for each parasite, the sex category and parasitism by *P. illiciens* (*p* < 0.0001) showed a strong association with a higher frequency of females parasitized by the fluke. According to Headley et al. (2011) and Gava et al. (2015), this strong association is related to the fact that females hunt more, mainly to feed their youth.

Still comparing the categories with each parasite, a strong correlation was observed between the age category and individuals parasitized by *Cystoisospora* spp. (*p* = 0.0014). With a decrease in cases with the increasing age of individuals, an immature immune system is a possible cause (Carrasco et al., 2016). The self-limiting nature of this parasite makes the results more understandable, since adult and elderly animals would have their immune systems already prepared to contain a reinfection.

With the analysis of the results of the present study, the association of fecal consistency and positive animals showed significance only for the parasites *G. intestinalis* (*p* = 0.007) and *P. illiciens* (*p* = 0.033), but for type 3 feces, which for the authors of the present study would be normal feces. Reinforcing the idea that the isolated evaluation of fecal consistency does not allow presuming the presence or absence of the parasite, since the feces can present alterations due to different factors (German et al., 2017) and many animals with normal stools are positive for parasite infection, being the correct diagnosis using the appropriate tools.

The coproparasitological research in the present study provides interesting data regarding the diagnosis of *Mesocestoides* spp., a zoonotic tapeworm of the family Mesocestoididae that parasitizes several carnivores. According to a survey of publications related to the parasite in question between 1940 and 2020 carried out by Chelladurai & Brewer (2021), this parasite was more frequent in wild carnivores, with few reports in domestic carnivores, such as cats. According to the authors, the low prevalence of this parasite is due to the restriction of the domestic cat to contact with infected intermediate hosts and thus their predation, with parasitism being more likely in stray animals. In addition to the low sensitivity of coproparasitological, sedimentation and/or fluctuation tests, which lead to underdiagnosis. This may explain the low diagnostic frequency of this helminth in the present study, as the samples were processed using flotation and/or sedimentation methods. However, another important factor observed in the study was the light coloration of this cestode (Figure 1F), allowing, in some cases, the non-visualization of the egg depending on the dirtiness of the examination and/or the intensity of the light focus of the microscope.

In the present study, CFS was not used to diagnose protozoa such as *G. intestinalis* and *T. gondii/H. hammondi* to avoid generating false results, as *G. intestinalis* cysts deform in solutions with high specific gravity, such as those used in this technique, and are confused with artifacts. As pointed out by Dryden et al. (2005), solutions with a high specific gravity allow many pseudoparasites/artifacts, such as yeast, plant remains, and debris, to be confused with these parasites. In addition, as suggested by the authors, there is a lack of microscopes that allow measurements at the time of reading.

With the use of different coproparasitological techniques, it was possible to broadly verify the main populations of parasites existing in the gastrointestinal system of cats without observing, in general, the difference between the sensitivity of the techniques (Table 3), with the exception of specificity for the parasites. There were significant differences in the diagnosis of diphyllobothrids (*p* = 0.0076), *P. illiciens* (*p* = 0.002), and *Cystoisospora* spp. (*p* = 0.0461).

Diagnoses of parasites of the family Diphyllobothriidae were only obtained using the spontaneous sedimentation technique, suggesting greater sensitivity for the diagnosis of cestodes of this family compared to other techniques. This corroborates the data reported by Dib et al. (2019), who compared different coproparasitological techniques in the diagnosis of *S. mansonoides* (Pseudophyllidea: Diphyllobothriidae) in wild mammals and observed greater sensitivity in sedimentation techniques than in flotation techniques.

The diagnosis of the trematode *P. illiciens* indicated a possible difference between the techniques, scoring better performance of the CFS and spontaneous sedimentation techniques when compared to that of Faust et al. (1939). A positive performance of the sedimentation technique was expected because it is indicated for the diagnosis of heavy eggs such as trematodes (Foreyt, 1989; Dib et al., 2019). However, the CFS technique, which is routinely used for the diagnosis of oocysts and helminth eggs, demonstrated excellent performance in the diagnosis of *P. illiciens*. This result is similar to that found by Rocha et al. (2014), who compared the CFS technique with centrifugal sedimentation in formalin-ether (the standard technique in the diagnosis of *P. illiciens*) and observed greater performance in the diagnosis of *P. illiciens* by CFS. Dib et al. (2019) explained that this occurred because the high density of sucrose solution allowed some sealed eggs to float.

Finally, regarding the diagnosis of *Cystoisospora* spp., it was possible to observe a better performance of the centrifugal flotation technique in relation to the sedimentation technique. However, even with parasitic structures diagnosed using flotation techniques (Foreyt, 1989), Rezende et al. (2015) and Lima et al. (2018) demonstrated higher sensitivity and specificity of the sedimentation technique in the diagnosis of *Cystoisospora* spp. A possible cause of the low diagnostic accuracy of the spontaneous sedimentation technique in this study may be related to the low parasite load of the evaluated samples.

In the present study, we demonstrated the presence of parasites in fecal samples from domestic cats, demonstrating important associations between parasitism and sex, age, and fecal consistency. Regarding coproparasitological techniques, it was possible to observe the importance of associating more than one type of technique for the diagnosis of different groups of parasites, allowing a broader and more reliable diagnosis.

## Conclusions

In conclusion, the use of CFS, centrifugal flotation in zinc sulfate, and spontaneous sedimentation techniques allowed the determination of the occurrence of parasites in fecal samples from domestic cats, with *Ancylostoma* spp. and *G. intestinalis* being the most frequent. In addition, the correlations between the evaluated categories suggest strong relationships of parasitism by *Cystoisospora* spp. in young animals, greater propensity for parasitism by *P. illiciens* in females and animals positive for *P. illiciens* and *G. intestinalis* infections associated to feces with normal consistency.
